# Surface-induced vibrational energy redistribution in methane/surface scattering depends on catalytic activity

**DOI:** 10.3389/fchem.2023.1238711

**Published:** 2023-07-25

**Authors:** Patrick Floß, Christopher S. Reilly, Daniel J. Auerbach, Rainer D. Beck

**Affiliations:** ^1^ Institute of Chemical Sciences and Engineering (ISIC), École Polytechnique Fédérale de Lausanne (EPFL), Lausanne, Switzerland; ^2^ Max Planck-EPFL Center for Molecular Nanoscience and Technology, Lausanne, Switzerland; ^**3**^ Max Planck Institute for Multidisciplinary Sciences, Göttingen, Germany

**Keywords:** methane dissociation, state-to-state scattering, angular distributions, surface-induced vibrational energy redistribution, optothermal spectroscopy, bolometer infrared laser tagging, heterogeneous catalysis

## Abstract

Recent state-to-state experiments of methane scattering from Ni(111) and graphene-covered Ni(111) combined with quantum mechanical simulations suggest an intriguing correlation between the surface-induced vibrational energy redistribution (SIVR) during the molecule/surface scattering event and the catalytic activity for methane dissociation of the target surface (Werdecker, Phys. Rev. Res., 2020, 2, 043251). Herein, we report new quantum state and angle-resolved measurements for methane scattering from Ni(111) and Au(111) probing the extent of $\nu_{3}\to \nu_{1}$ antisymmetric-to-symmetric conversion of methane stretching motion for two surfaces with different catalytic activities. Consistent with the expectations, the extent of SIVR occurring on the more catalytically active Ni(111) surface, as measured by the $\nu_{1}:\nu_{3}$ scattered population ratio, is found to be several times stronger than that on the more inert Au(111) surface. We also present additional insights on the rovibrational scattering dynamics contained in the angle- and state-resolved data. The results together highlight the power of state-resolved scattering measurements as a tool for investigating methane–surface interactions.

## 1 Introduction

State-to-state scattering experiments provide a powerful and well-established technique to elucidate the atomic-level details of what happens when molecules collide with other gas-phase molecules or with surfaces (Auerbach et al., 2021). Often, the connection between the measured final state distributions and the property under study is direct and obvious. For example, the degree of rotational excitation in a collision is directly related to the angular anisotropy of the potential energy surface (PES) of the system under study and can be used to test theoretical methods for calculating the PES. In other cases, the connection of the scattering data to the underlying question of what is going on is more subtle. For example, highly vibrationally excited NO or CO molecules scatter almost vibrationally elastically from insulator surfaces but undergo facile multi-quantum vibrational relaxation if they strike a metal surface. Qualitatively, these observations show that the presence of a continuum of low-lying electronically excited states in the metal is being excited by molecular vibrations-a strong violation of the Born–Oppenheimer approximation. More detailed theoretical work established that charge transfer in the collisions is the key driving factor and that the propensity for charge transfer therefore correlates with the degree of vibrational relaxation (Wodtke et al., 2008; Shenvi et al., 2009; Wagner et al., 2019).

An intriguing example of this more subtle connection of observations to dynamical propensities emerges from the first state-to-state measurements of methane scattering from surfaces. When CH_4_ ( $\left.\nu_{3}=1,J=1\right)$ , that is, CH_4_ prepared with a single vibrational quantum in the $\nu_{3}$ antisymmetric C-H stretch normal mode and one quantum of vibrational angular momentum, strikes a clean Ni(111) surface, nearly 40% of the molecules scatter into a state with one quantum of the $\nu_{1}$ symmetric C-H stretch normal mode (Werdecker et al., 2018). In this study, we refer to this phenomenon of surface collision-mediated conversion of vibrational energy by the term *surface-induced vibrational energy redistribution*, or SIVR for short. We note that a similar SIVR process for scattering of H_2_O from Cu(111) was predicted by state-to-state quantum scattering calculations by Zhang and Jiang (2019) and Zhang and Jiang (2020).

Surprisingly, SIVR was not observed for CH_4_ ( $\nu_{3}=1,J=1$ ) scattering from Gr/Ni(111), the graphene-covered Ni(111) surface (Werdecker et al., 2020) (for convenience we refer to reference [Werdecker et al., 2020] from now on as “*Werdecker et al.*”). Quantum dynamics calculations by Bret Jackson and coworkers propose an explanation for this remarkable difference. Their calculations show that as the incident CH_4_ ( $\nu_{3}$ ) molecules approach the reactive Ni(111) surface, the C-H bond closest to the surface starts to elongate, introducing a coupling between the two C-H stretching normal modes $\nu_{3}$ and $\nu_{1}$ which differ only by the relative phases of the C-H stretching motions. Inelastic scattering calculations for collisions with different surface sites on Ni(111) reveal a correlation between the calculated C-H bond elongation and the catalytic activity of the surface site, resulting in an increasing probability for SIVR with increasing catalytic activity of the impact site. Based on these results, the authors hypothesized that the extent of SIVR in a molecule–surface collision is correlated with the catalytic activity of the surface.

In this paper, we present new measurements to test this hypothesis. We extend the range of surfaces studied to include the closely packed Au(111) surface which, based on density function calculations^1^ of the barrier to dissociative adsorption, is expected to have a catalytic activity intermediate to that of Ni(111) (Jackson and Nave, 2013) and Gr/Ni(111) (Li et al., 2018).

In addition to providing data on a new system with catalytic activity intermediate between Ni(111) and Gr/Ni(111), we have also improved the method of determining the “branching ratio” $br_{\nu_{1}/\nu_{3}}$ , which quantifies the extent of SIVR through the ratio of the total $\nu_{1}$ and $\nu_{3}$ scattering flux following the surface collision of an incident CH_4_ ( $\nu_{3}$ ) molecule. We use a rotatable bolometer detector in this work, which allows us to measure quantum state-resolved angular distributions of scattered molecules. We base our reported values of $br_{\nu_{1}/\nu_{3}}$ on the scattering angle- and rotational state-integrated scattered flux of CH_4_ in the $\nu_{1}$ and $\nu_{3}$ states. Previous determinations of $br_{\nu_{1}/\nu_{3}}$ were based on measurements at a single scattering angle due to limitations of the instrument employed in those studies. Indeed, as shown in Section 3.2, the angular distributions differ for the different scattered CH_4_ rovibrational states probed. Therefore, for the measured branching ratio to reflect the total $\nu_{3}\to \nu_{1}$ and $\nu_{3}\to \nu_{3}$ scattering probabilities accurately, it is advantageous to measure over a wide range of scattering angles and integrate the $\nu_{1}$ and $\nu_{3}$ scattered intensities over the scattering angle.

Our measurements show that the value of $br_{\nu_{1}/\nu_{3}}$ measured for Au(111) is intermediate to those of Ni(111) and Gr/Ni(111). The literature values of the activation barriers to dissociative chemisorption increase when proceeding from Ni(111) (Jackson and Nave, 2013) to Au(111) (Jackson) to (free-standing) graphene (Li et al., 2018). Thus, our results are in accordance with the hypothesis that SIVR correlates with the catalytic activity of the surface.

## 2 Materials and methods

**FIGURE 1:**
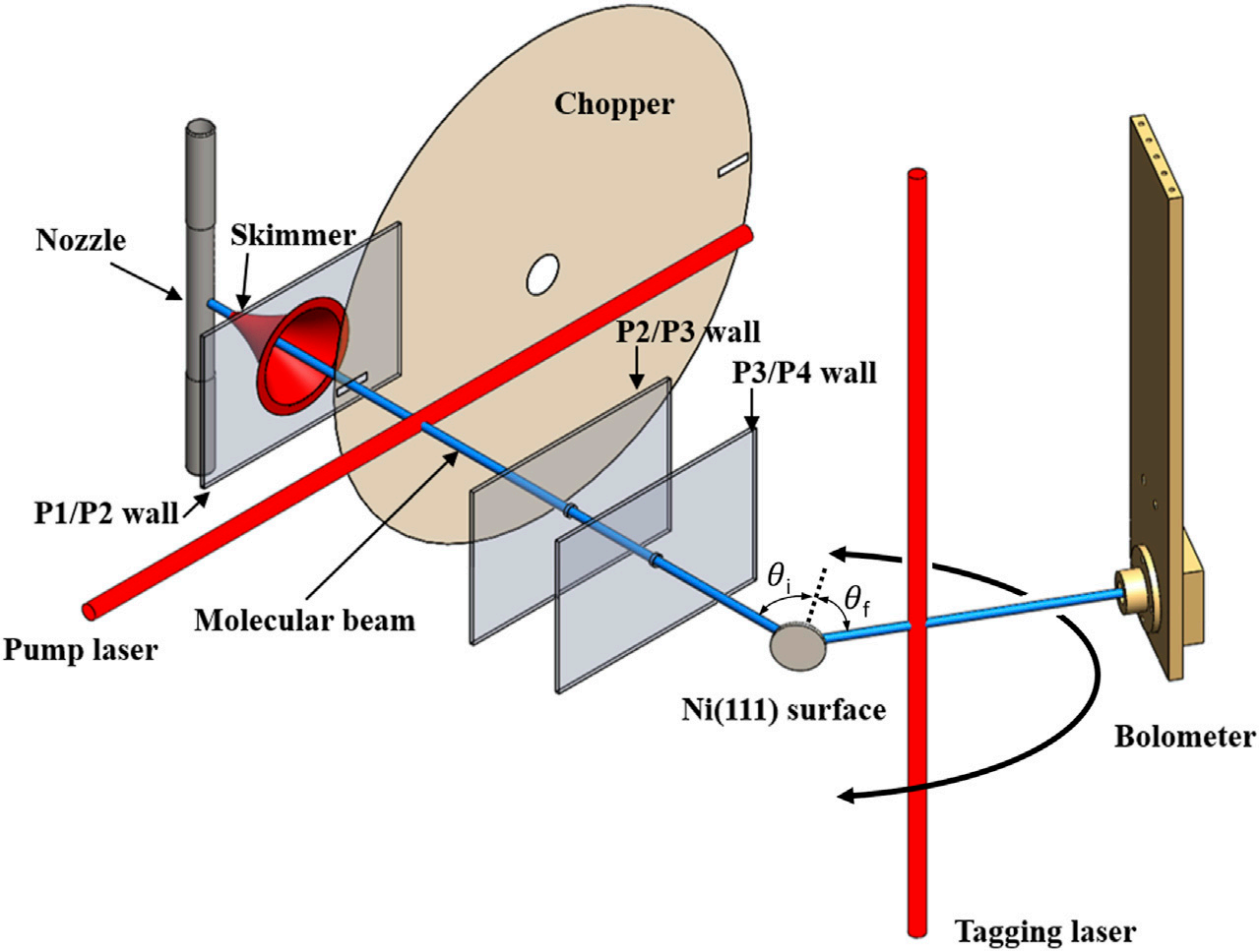
Schematic of the experimental setup used for state-to-state surface scattering of methane. A molecular beam is generated in vacuum chamber P1 and traverses the differential pumping stages P2 and P3. Quantum state-specific preparation of methane in the molecular beam occurs in P2 which also contains a chopper disk for beam modulation. The state-prepared molecular beam collides in P4 with the single crystal target surface at an incident angle $\theta_{i}$ with respect to the surface normal (marked by the dotted line). Molecules scattered at an angle $\theta_{f}$ are excited with quantum state resolution by the tagging laser and detected by the rotatable bolometer.

Figure 1 shows a schematic illustration of the experimental setup. Full details can be found in Reilly et al. (2023). Briefly, a continuous supersonic expansion from a temperature-controlled nozzle into a vacuum is used to create a molecular beam of methane with the mean kinetic energy of 100 meV and a FWHM spread of 30%. Approximately 10% of the methane molecules in the molecular beam are prepared in the J=1 *meta*-CH_4_ (i.e., nuclear spin I=2 ) rotational state of the $\nu_{3}$ antisymmetric stretch fundamental (i.e., with one quantum of $\nu_{3}$ vibration) by infrared laser excitation with a tunable, single-mode, continuous-wave (CW) infrared (IR) optical parametric oscillator (OPO) (Argos Lockheed Martin-Aculight, “Pump laser” in Figure 1) (Chadwick et al., 2014). The remaining ≈90% of the molecules populate rotational states in the ground vibrational state not addressed by the pump laser.

The state-prepared molecular beam collides with a single crystal surface sample in the ultrahigh vacuum surface science chamber with a base pressure of 1 × 10^−10^ mbar. A 4-axis manipulator permits displacement the surface in three dimensions and rotation of the incident angle $\theta_{i}$ made between the surface normal and the molecular beam.

Scattered molecules are detected and resolved by the quantum state and scattering angle using the bolometer infrared laser tagging (BILT) technique (Reilly et al., 2023). Here, a second, tunable, single-mode, CW IR OPO (TOPO TOPTICA Photonics, “tagging laser” in Figure 1) laser beam is chopped at 237 Hz with 50% duty cycle using an optical chopper wheel (not shown) and crosses the scattering plane perpendicularly 16 mm from the target surface. By tuning the tagging laser into resonance with different rovibrational transitions, we selectively excite the methane molecules that have scattered from the initially prepared state into the lower state of the resonant transition, transferring to those molecules one quantum of $\nu_{3}$ vibrational energy. The approximately 370 meV/molecule of energy absorbed by the molecules from the tagging laser is then transferred to a cryogenic bolometer detector when the molecules adsorb on a cold 4-mm-diameter diamond absorber to which the bolometer is attached.

Using a lock-in amplifier, we record the response of the detector to the chopping of the tagging laser. To the raw lock-in output, we apply a series of corrections to obtain a “scattering intensity” $s\left(\theta_{f}\right)$ which is intended to provide a relative measure of the fraction of molecules scattering into the range of solid angles subtended by the bolometer detector centered at an angle $\theta_{f}$ in the scattering plane and occupying the state being tagged. These corrections are described in detail in Section S1 of the Supplementary Material of Reilly et al. (2023).

**FIGURE 2:**
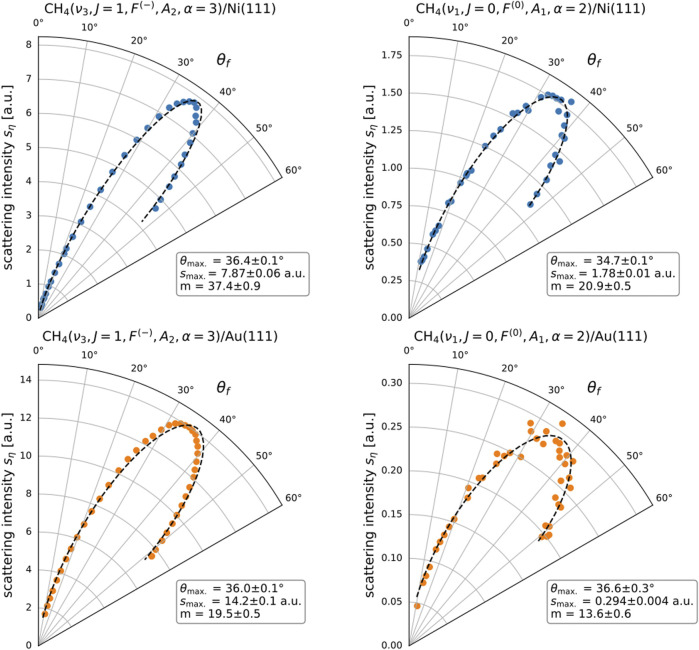
State-resolved in-plane angular distributions $s_{\eta }\left(\theta_{f}\right)$ for CH_4_( $\nu_{3},J=1,F^{\left(-\right)},A_{2},\alpha =3$ ) scattering from Ni(111) (blue markers) and Au(111) (orange markers). The incoming molecules have a mean kinetic energy $E_{i}=100\pm 13meV$ and strike the surface ( $T_{s}=473K$ ) at an incident angle $\theta_{i}=35^{\circ }$ . The points correspond to measured data, and the dashed lines correspond to the associated fits (see Eq. 1). The best-fit parameter values are listed in the boxes next to their associated plots. The main text shows the explanation of quantum state identifiers ( $\nu_{a}$ , J , $F^{\left(b\right)}$ , $A_{c}$ , and α ). Reported scattering intensities are obtained from raw detector signals after the application of corrections for tagging efficiency and time-dependent bolometer sensitivity and represent the differential scattered flux (in molecules per steradian per second) within a multiplicative constant independent of the surface and rovibrational state. The absolute value of the angle between the surface projection of the molecule’s initial velocity and the $\left\{\bar{1}2\bar{1}\right\}$ crystallographic direction of the Ni(111) and Au(111) surface samples is $10^{\circ }\pm 2^{\circ }$ and $1^{\circ }\pm 2^{\circ }$ , respectively.

Synchronized, computer-controlled rotation of the bolometer and the tagging laser beam enables variation of the measured final angle $\theta_{f}$ , enabling, in turn, measurement of the state-resolved angular distributions of scattered methane molecules, as shown in Figure 2 in the following section.

For preparation of atomically flat Ni(111) and Au(111) surfaces, we use Ar^+^ sputtering followed by annealing to 973 K and 773 K, respectively. Crystallographic order of prepared surfaces is verified using low-energy electron diffraction (LEED), and the absence of surface contaminants is verified using Auger electron spectroscopy. After conducting all experiments, the contamination levels were verified to be below the detection threshold of approximately 1% ML.

## 3 Results and discussion

For both the Ni(111) and Au(111) surfaces, we recorded 14 state-resolved angular distributions. To identify the rovibrational level being tagged, we use the quantum numbers ( $\nu_{a}$ , J , $F^{\left(b\right)}$ , $A_{c}$ , and α ) which, for brevity, we sometimes condense down to a single compound index η . $\nu_{a}$ specifies the symmetry ( a=1 for symmetric and a=3 for antisymmetric) of the stretch fundamental (or “stretch” for simplicity), J denotes the total angular momentum, $F^{\left(b\right)}$ denotes the Coriolis label ( b=−,0,+ ) characterizing the coupling of rotational and vibrational angular momentum (di Lauro, 2020), $A_{c}$ denotes the wavefunction symmetry ( c=1,2 ), and α denotes the polyad-level identifier (Brown et al., 1992). The rovibrational level prepared by the pump laser, for example, has quantum numbers ( $\nu_{3},J=1,F^{\left(-\right)},A_{2},and\;\alpha =3$ ). Note that for the subset of levels tagged in our study, the first four identifiers (i.e., $\nu_{a}$ , J , $F^{\left(b\right)}$ , and $A_{c}$ ) suffice to uniquely identify a level.

Figure 2 illustrates a selected subset of the measured angular distributions. The complete set of measured distributions is available in Supplementary Section S1. The rotational states tagged include all *meta*-CH_4_ states in the $\nu_{3}$ and $\nu_{1}$ stretch fundamentals with total angular momentum quantum number J≤5 along with one J=7 rotational level for both stretches. States with nuclear spin I≠2 were not probed because interconversion among the different nuclear spin isomers in direct molecule–surface collisions is presumed to occur with negligible probability. Our own measurements using a double-resonance pump–probe technique showed no evidence of such interconversion in CH_4_/Au(111) collisions.

In the following sections, we present an analysis and discussion of the measured distributions. First, in Section 3.1, we treat the central topic of SIVR before moving on to a detailed look at aspects of scattering dynamics relating to the scattering angle (Section 3.2) and rotational state (Section 3.3).

### 3.1 Surface-induced vibrational energy redistribution

The principle objective of the current work is to test the hypothesis advanced in *Werdecker et al.* and discussed in the Introduction of the current work. We remind the reader that this hypothesis asserts a positive correlation between the efficacy of $\nu_{3}\to \nu_{1}$ conversion induced in a methane–surface collision and the catalytic activity of the surface for the dissociative chemisorption of methane. To quantify the efficacy, we compute a branching ratio $br_{\nu_{1}/\nu_{3}}$ using the following procedure. For each tagged rovibrational state η , we integrate the measured scattering intensity $s_{\eta }\left(\theta_{f}\right)$ over the range ${11.5^{\circ }=\theta }_{-}<{\theta_{f}<\theta }_{+}=47^{\circ }$ of angles probed and then sum the resulting integrated fluxes $S_{\eta }=\int_{\theta_{-}}^{\theta_{+}}s_{\eta }\left(\theta_{f}\right)d\theta_{f}$ over the different tagged rotational states of each stretch. We obtain a measure of the total scattered flux ${\bar{S}}_{\nu_{a}}=\underset{\eta \in \nu_{a}}{\sum }S_{\eta }$ of each stretch fundamental $\nu_{a}$ . From ${\bar{S}}_{\nu_{1}}$ and ${\bar{S}}_{\nu_{3}}$ , we obtain the branching ratio $br_{\nu_{1}/\nu_{3}}={\bar{S}}_{\nu_{1}}/{\bar{S}}_{\nu_{3}}$ .

The branching ratios $br_{\nu_{1}/\nu_{3}}$ obtained from the state-resolved angular distributions are shown in Table 1, along with the branching ratio of zero implied from the results of *Werdecker et al.* in their fixed scattering angle measurements for the graphene-covered nickel surface. Tabulated along with the branching ratios are the associated barrier heights for dissociative chemisorption of CH_4_ as determined by density functional theory (DFT) calculations. We note that the barrier height quoted for Gr/Ni(111) was in fact computed for free-standing graphene, although the addition of a Ni(111) substrate is not expected to alter this figure significantly^1^. Taking a low barrier height as an indication of high catalytic activity, it is clear that the order of the branching ratios and barrier heights for these three surfaces agrees with the stated hypothesis.

**TABLE 1:** Scattering angle-integrated branching ratios $br_{\nu_{1}/\nu_{3}}$ computed from the state-resolved angular distributions measured in this study (Figure 2) along with theoretically calculated barrier heights for methane dissociation with associated references.

Surface	$br_{\nu_{1}/\nu_{3}}$ (%)	Barrier height (eV)
Ni(111)	35.1 ±4	1.1^10^
Au(111)	6.6 ±0.7	1.9^9^
Gr/Ni(111)	0.0^†^	4.1^‡11^

^†^From reference [Werdecker et al., 2020]. Measured under conditions $E_{i}=100\;meV$ , $T_{s}=673K$ , $\theta_{i}=65^{\circ }$ , and fixed $\theta_{f}=70^{\circ }$ .

^‡^Calculated for free-standing graphene.

To assess the repeatability of the branching ratio measurements the full set of measurements for the Au(111) surface was performed twice. The data from the second run showed a somewhat better signal-to-noise ratio and so we present the branching ratio of 6.6% obtained from this run in Table 1. The first run yielded a branching ratio of 7.9%. The percentage deviation of $100\%\times \frac{7.9\%-6.6\%}{7.9\%+6.6\%}=9\%$ between runs is of the same order of magnitude as those measured for other quantities derived from the same runs (e.g., the mean rotational energies $\langle E_{rot.}^{\nu_{1}}\rangle$ and $\langle E_{rot.}^{\nu_{3}}\rangle$ presented in Section 3.3). As there is a lack of necessary data for a more rigorous statistical analysis, we take ±10% as a reasonable estimate of our branching ratio uncertainty for the Ni(111) surface as well as the Au(111) surface, with the experimental procedures and data quality for the two surfaces being largely identical. From our own analysis of the signal-to-noise ratio of the measurements presented in *Werdecker et al.* on Gr/Ni(111), we arrive at a rough estimate of the upper limit of the branching ratio for this surface of 2%. We therefore consider the branching ratio order presented in Table 1 to be reliable.

### 3.2 Trends in state-resolved angular distributions

In computing the branching ratios, we integrate over the scattering angle and sum over rotational states and thus discard any dynamical information contained in the angular and rotational state dependence of the scattering intensities. To explore in finer detail the dynamics of the molecule–surface collision, we return in this section to the state-resolved angular distributions. In the following section (Section 3.3), we narrow our attention to the gross rotational dynamics via the scattering angle-integrated state fluxes $S_{\eta }$ .

We have found it useful to summarize individual state-resolved angular distributions by three parameters, namely, m , $\theta_{\max.}$ , and $s_{\max.}$ , which characterize a distribution’s width, angle of peak intensity, and peak amplitude, respectively. The parameters are determined by performing a non-linear least squares fit of the measured data using a fit function of the following form:

$$s\left(\theta_{f}\right)=s_{\max.}\cdot \cos^{m}\left(\theta_{f}-\theta_{\max.}\right)$$ (1)

That the chosen fit function gives a good description of our observations can be verified by inspection of Figure 2 which shows measured data overlaid with their associated best-fit curves (see also the Supplementary Materials for full set of angular distributions and fits).

**FIGURE 3:**
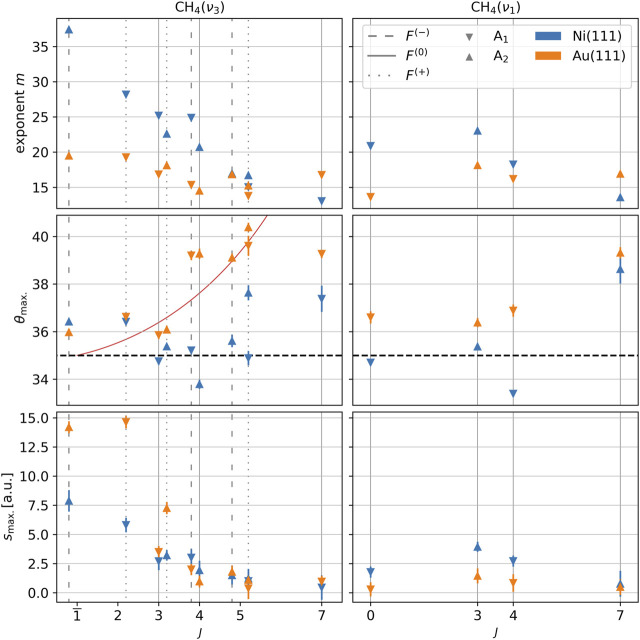
Fitting parameters obtained from fitting Eq. 1 to the measured state-resolved in-plane angular distributions $s_{\eta }\left(\theta_{f}\right)$ . The rovibrational level $\left(\nu_{3},J=1,F^{\left(-\right)},A_{2}\right)$ prepared by the pump laser is indicated by the overbar $\bar{1}$ on the J label. Tagged levels are grouped by the total angular momentum quantum number J and displaced slightly left, right, or not at all depending on whether their associated Coriolis stack is $F^{\left(-\right)}$ , $F^{\left(+\right)}$ , or $F^{\left(0\right)}$ , respectively. Marker symbols denote the wavefunction symmetry. The red curve in the plot of $\theta_{\max.}$ for the $\nu_{3}$ levels is the predicted $\theta_{\max.}$ for a rigid methane molecule with J=1 scattering from a flat and rigid surface. The two black dotted horizontal lines in the $\theta_{\max.}$ plots mark the angle of specular scattering.

Figure 3 shows the best-fit parameter values m , $\theta_{\max.}$ , and $s_{\max.}$ for all 28 state-resolved measurements (although we include in Figure 3 the peak amplitude parameter $s_{\max.}$ for completeness, we omit discussion of this parameter, deferring discussion of the related scattering angle-integrated state populations $S_{\eta }$ for Section 3.3). From the figure, one can readily deduce that for both surfaces and for all lines tagged the scattering mechanism appears to be “direct” or “impulsive” in that the measured distributions strongly deviate from the $m=1,\theta_{\max.}=0^{\circ }$ behavior expected of a “trapping/desorption” scattering process characterized by an extended molecular residence time at the surface (Rettner and Auerbach, 1994). Such a lack of equilibration between the surface and the scattering methane molecule has been seen before both in experiment and calculations for Ni(111) (Milot et al., 2001; Al Taleb and Farías, 2017) and Pt (111) (Kondo et al., 2018).

In addition to our classification of the scattering as direct/impulsive, we also identify three distinct trends from the analysis of the angular distributions. First, the exponents m (Figure 3, top row) are systematically higher for scattering from Ni(111) than those from Au(111), reflecting a lower degree of angular dispersion caused by the former. Second, for both Ni(111) and Au(111), the widths of the scattering peaks increase with the rotational quantum number J , suggesting a correlation between rotational excitation and momentum transfer either between the molecule and the surface or between the parallel and perpendicular components of the molecular momentum.

Third, the peak angles $\theta_{\max.}$ (Figure 3, middle row) for scattering from Au(111) are shifted further away from surface normal compared to those for scattering from Ni(111). In a recent publication, we reported (Reilly et al., 2023) the same finding for CH_4_/Ni(111) scattering with CH_4_ prepared in the vibrational ground state and noted in addition a positive trend between $\theta_{\max.}$ and total angular momentum quantum number J . From Figure 3, it is clear that this correlation holds for the vibrationally excited molecules as well, the trend being somewhat more pronounced for Au(111). Note that a positive $\theta_{\max.}$ – J correlation is what one anticipates for the scattering of a rigid body from a flat and rigid surface based on simple considerations of conservation of energy and parallel momentum (see the red curve of Figure 3). One might therefore conclude that the CH_4_/Au (111) system more closely approaches this ideal than the CH_4_/Ni(111) system. However, in the limit of perfect flatness and rigidity, one also expects a unique scattering angle for each final state (i.e., m→∞) . On this account, the broader peaks (characterized by smaller exponents m ) observed for scattering from Au(111) would then imply the opposite conclusion. Our results do not therefore admit a conclusive determination of which surface appears more “mirror-like” to a scattering methane molecule. Of course, a vibrating molecule cannot in the first place be said to be a rigid body, and certainly for the $\nu_{3}\to \nu_{1}$ scattering channel, the situation is further complicated by the change in the vibrational mode structure and accompanying energy release.

Going beyond the trends just discussed, we can also make a few additional qualitative observations. We first point out the anomalously large exponent m for the elastic $\left(\nu_{3},J=1\right)\to \left(\nu_{3},J=1\right)$ channel on Ni(111) is 38% larger than the next-largest $\nu_{3}$ channel exponent and 61% larger than the largest $\nu_{1}$ exponent for the same surface. In a practical sense, this fact, combined with the fact that the elastic channel on Ni(111) is relatively intense, establishes that the angular distributions for the vibrationally elastic and inelastic channels can in fact differ and that it is, therefore, essential to measure overall scattered angles to obtain the most accurate estimate of the total $\nu_{3}\to \nu_{1}$ scattering probability. Indeed, when we compute “fixed angle branching ratios” $\underset{\eta \in \nu_{1}}{\sum }s_{\eta }\left(\theta_{f}\right)/\underset{\eta^{\prime }\in \nu_{3}}{\sum }s_{\eta^{\prime }}\left(\theta_{f}\right)$ from our Ni(111) measurements, we find that their values vary by more than 50% over the range of scattering angles $\theta_{f}$ probed (Supplementary Section S2).

In another sense, that the vibrationally and rotationally elastic scattering peak is so much narrower than the others and should imply something about the molecule–surface interaction potential and associated scattering dynamics. We offer the following simple microscopic explanation for a strong and anomalously narrow elastic scattering distribution peaking near specular ( $\theta_{\max.}-\theta_{i}<1.5^{\circ }$ ). Of the full range of values of impact parameters characterizing the possible initial conditions of the scattering molecule (e.g., impact site, initial molecular orientation, and internal/external axis of rotation), there is a significant fraction which results in trajectories where the molecule is turned around at distances relatively far from the surface. At these distances, the interaction between the molecule and the surface manifests primarily as a mutually repulsive force directed along the surface normal. That is, for these trajectories, the molecules do not penetrate far enough for the molecule–surface interaction to either exhibit significant lateral surface corrugation or effect significant disruption of the molecule’s or surface’s internal structure. Thus, the various mechanisms available to induce a change in the rovibrational state or deflect the scattering molecule away from specular scattering (parallel-to-perpendicular momentum transfer, surface phonon creation/annihilation, and rovibrational inelasticity) would act only weakly, leading to a narrow elastic distribution peaking near specular scattering. On the other hand, trajectories permitting deep enough penetration for the interaction to effect change in the rovibrational state will also tend to disperse angularly due to the additional effects of lateral corrugation and phonon creation/annihilation, resulting in broader scattering distributions for the rovibrationally inelastic channels. Interestingly, the strength of these dispersive forces seems to be no greater for the trajectories undergoing vibrational state change than those simply changing rotational state.

To qualitatively summarize the explanation just presented, our observations can be interpreted as implying a certain order in which the different aspects of the molecule–surface interaction “turn on” as the molecule approaches the surface, with a two-body repulsive force preceding the onset of dispersive forces, which in turn precedes the onset of forces acting to distort or rotate the molecule.

We close the section with a discussion regarding the differences in peak widths $\propto 1/\sqrt{m}$ and peak angles $\theta_{\max.}$ , particularly for the Ni(111) data, among the levels of equal *J* in the $\nu_{3}$ stretch. These levels have the same nominal rotational energy (within ±2% ^20^) but are distinguished by their associated Coriolis stack $F^{\left(b\right)}$ and wavefunction symmetry $A_{c}$ . The Coriolis stack of a level indicates, loosely speaking, the relative orientation of its vibrational angular momentum and its total angular momentum (parallel, perpendicular, or antiparallel for $F^{\left(-\right)}$ , $F^{\left(0\right)}$ , and $F^{\left(+\right)}$ , respectively) (di Lauro, 2020). Centrifugal coupling between rotation and vibration splits a Coriolis level ( ±0.3% worst case (Hecht, 1961a)) into “sublevels” of definite symmetry with respect to the $T_{d}$ point group of the CH_4_ molecules (Hecht, 1961b), of which those of $A_{1}$ and $A_{2}$ symmetries belong to the *meta*-CH_4_ nuclear spin isomer. The sublevels of a Coriolis stack are distinguished by the internal alignment of their total angular momentum, i.e., by the different molecule-fixed axes about which the molecule is more and less likely to be found rotating (again, loosely speaking).

Compare for example the best-fit m and $\theta_{\max.}$ values for the ( $\nu_{3},J=4,F^{\left(-\right)},A_{1}$ ) and ( $\nu_{3},J=4,F^{\left(0\right)},A_{2}$ ) levels scattering from Ni(111). The peak of the $F^{\left(0\right)}$ level has a 25% larger best-fit exponent m and a peak angle $\theta_{\max.}$ $1.5^{\circ }$ closer to normal than that of the $F^{\left(-\right)}$ level. Similar differences occur between the $A_{1}$ and $A_{2}$ sublevels in the same ( $\left.\nu_{3},J=5,F^{\left(+\right)}\right)$ Coriolis stack. These rather subtle details of the internal molecular motion have a significant impact on the scattering distributions and highlight the “dynamic” nature of the CH_4_/Ni(111) interaction in that the form of the state-resolved distributions are not solely dictated by the state’s energy. In a recent publication published by the authors on surface scattering of CH_4_ in the vibrational ground state (Reilly et al., 2023), we found strong evidence for a more extensive molecule–surface collisional energy exchange (i.e., equilibration) after oxidation of a Ni(111) surface than before oxidation. We also found that the forms (and amplitudes) of the sublevel angular distributions (the ground vibrational state has only $F^{\left(0\right)}$ Coriolis stacks) produced upon scattering from the oxidized surface were essentially indistinguishable, while scattering from the unoxidized surface produced differences in the peak widths and peak angles for the different sublevels comparable to those observed in the $\nu_{3}$ vibrationally excited state. We argued that the lack of imbalances observed in the collisions with the oxidized surface was consistent with the other reported observations in that they reflected an equilibration among the sublevels caused by a prolonged molecule–surface energetic coupling acting to scramble any delicate internal rotational motion.

Concerning the Au(111) surface data, while the form of the distributions among $\nu_{3}$ levels of equal J is similar, we will see in the following section (Section 3.3) that the level populations $S_{\eta }$ (related indirectly to the peak amplitudes $s_{\max.}$ ) differ dramatically depending on the internal alignment of the molecule’s total and vibrational angular momentum, so that the scattering for this system is, in this sense, no less “dynamic.”

### 3.3 Rotational dynamics

**FIGURE 4:**
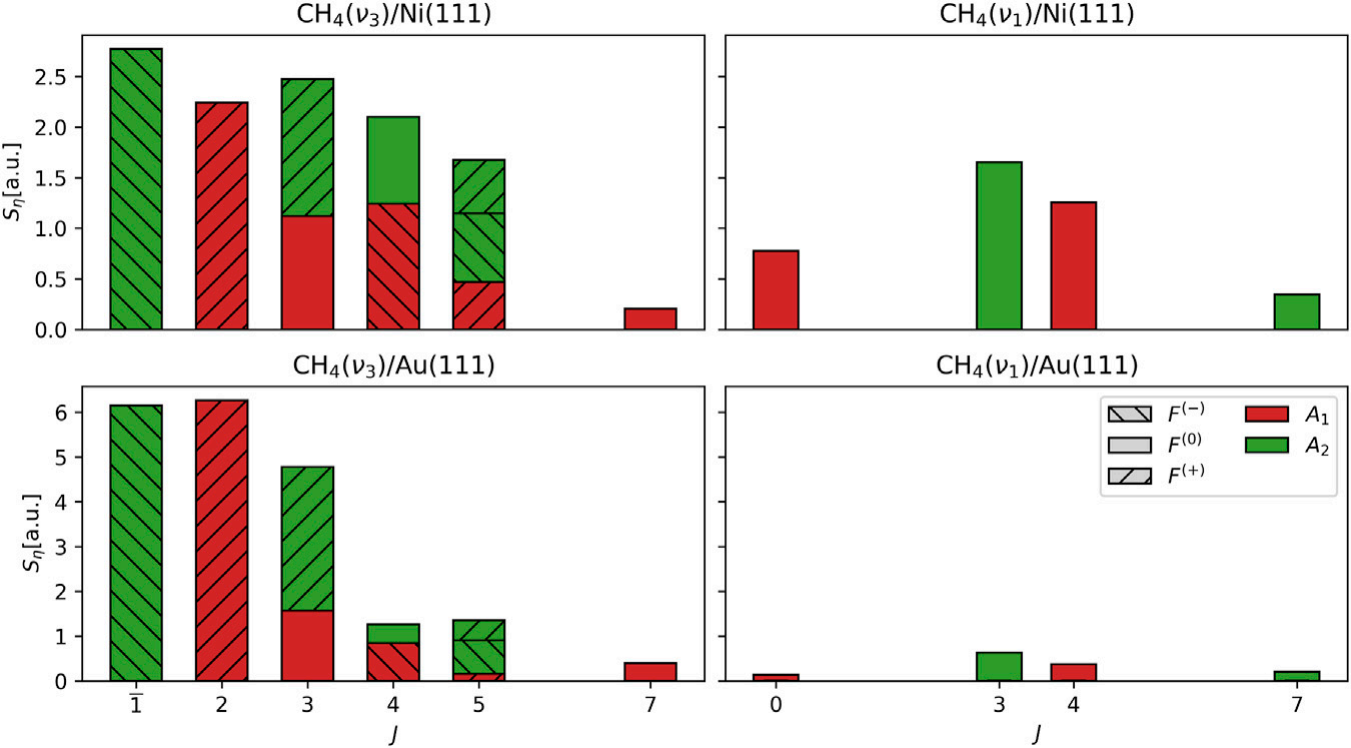
Distribution of scattering angle-integrated rovibrational state fluxes $S_{\eta }$ , where $\eta =\left(\nu_{a},J,F^{\left(b\right)},A_{c}\right)$ . The rovibrational level $\left(\nu_{3},J=1,F^{\left(-\right)},A_{2}\right)$ prepared by the pump laser is indicated by the overbar $\bar{1}$ on the J label. The Coriolis stack ( $F^{\left(-\right)}$ , $F^{\left(0\right)}$ , and $F^{\left(+\right)}$ ) of a tagged level is indicated by the hatching pattern of its associated bar and the wavefunction symmetry ( $A_{1}$ and $A_{2}$ ) by the bar color. All $\nu_{1}$ states are of the $F^{\left(0\right)}$ type. The weakly populated ( $\nu_{3}$ , J=5 , $A_{1}$ ) level for the Au(111) data is of the $F^{\left(+\right)}$ type (same as for Ni(111)).

Figure 4 shows the scattering angle-integrated rovibrational state populations $S_{\eta }$ . In addition to illustrating what is already evident from Table 1—namely, that the overall $\nu_{3}\to \nu_{1}$ conversion is much weaker for the Au(111) surface—the plotted distributions also reveal important additional features of the rotational dynamics of the molecular–surface collisions for the systems studied here.

One feature that is directly observable is the differences in the forms of the distributions of the vibrationally elastic ( $\nu_{3}\to \nu_{3}$ ) channels for the two surfaces, with the Au(111) distribution having a relatively narrow distribution concentrated at low J , suggesting a “colder” rotational population. To quantify the degree of rotational excitation in the different stretch fundamentals, we use two different measures. The first measure is the mean rotational energy $\langle E_{rot.}^{\nu_{a}}\rangle =\underset{\eta \in \nu_{a}}{\sum }S_{\eta }\varepsilon_{\eta }/{\bar{S}}_{\nu_{a}}$ obtained by taking the $S_{\eta }$ -weighted average of the rotational energies $\varepsilon_{\eta }$ of the different tagged levels η of the associated stretch $\nu_{a}$ . The rotational energy $\varepsilon_{\eta }$ of a level $\eta =\left(\nu_{a},J,F^{\left(b\right)},A_{c}\right)$ is calculated by taking the energy difference $\Delta \varepsilon_{\eta }^{o}$ between the level η and the rovibrational ground state as determined by high resolution spectroscopy (Gordon et al., 2022) and subtracting the theoretically determined energy gap $\Delta \varepsilon_{\nu_{a}}$ separating the $\nu_{a}$ vibrationally excited state from the ground vibrational state of a hypothetical non-rotating methane molecule (Boudon et al., 2006). The second measure is the effective rotational temperature $T_{rot.}^{\nu_{a}}$ obtained by fitting the $S_{\eta }$ of the associated stretch $\nu_{a}$ to a thermal (Boltzmann) distribution of the form Sη∝gηe−εηkTrot.νa , where k is the Boltzmann constant and $g_{\eta }=2J+1$ is the level degeneracy.

**FIGURE 5:**
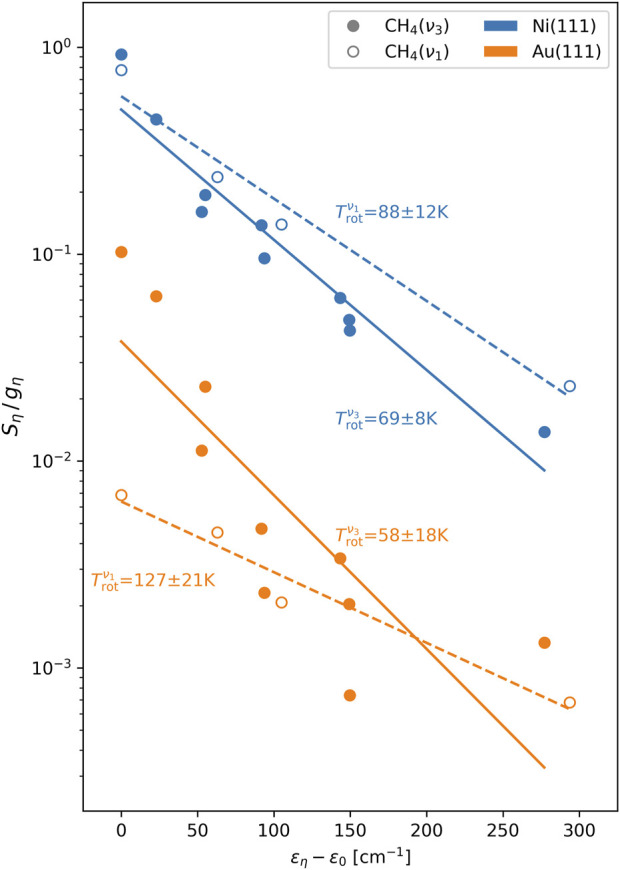
Boltzmann fits to the rotational-level population. Plotted are the scattering angle-integrated state-resolved fluxes $S_{\eta }$ weighted by the level degeneracy $g_{\eta }=2J+1$ *vs.* level energy $\varepsilon_{\eta }$ (within a constant $\nu_{a}$ -dependent shift $\varepsilon_{0}$ ). The fluxes $S_{\eta }$ for the Au(111) surface data have been scaled down by a factor 20 to avoid overlap with the Ni(111) data. Solid (dashed) lines indicate fits of the $\nu_{3}$ ( $\nu_{1}$ ) data to the Boltzmann form $\propto \exp\left(-\varepsilon_{\eta }/kT_{rot.}^{\nu_{3}\;\left(\nu_{1}\right)}\right)$ . Best-fit effective rotational temperatures $T_{rot.}^{\nu_{a}}$ are printed next to the associated best-fit line. The rovibrational level $\eta =\left(\nu_{3},J=1,F^{\left(-\right)},A_{2}\right)$ prepared by the pump laser corresponds to the left-most solid markers. The levels of a given stretch $v_{a}$ and total angular momentum J are nominally isoenergetic ( $\Delta \varepsilon_{\eta }<7{cm}^{-1}$ ), with the energy ordering for the different Coriolis stacks of a given $\left(\nu_{a},J\right)$ combination being $F^{\left(-\right)}<F^{\left(0\right)}<F^{\left(+\right)}$ . For both surfaces, the $A_{2}$ sublevel of the $\left(\nu_{3},J=5,F^{\left(+\right)}\right)$ Coriolis stack is more populated than the $A_{1}$ sublevel.

The extracted mean rotational energies $\langle E_{rot.}^{\nu_{a}}\rangle$ and effective rotational temperatures $T_{rot.}^{\nu_{a}}$ (Figure 5 for fits) are presented in Table 2. The differences in the $\nu_{3}$ distributions mentioned previously are reflected in the mean energies, with the $\langle E_{rot.}^{\nu_{3}}\rangle$ value for Ni(111) being 39% larger than that of Au(111). The difference in effective temperatures $T_{rot.}^{\nu_{3}}$ is less pronounced, due to the greater influence of the enhanced J=7 level population scattering from the Au(111) surface (level populations contribute linearly to $\langle E_{rot.}^{\nu_{a}}\rangle$ , while the $T_{rot.}^{\nu_{a}}$ fitting procedure minimizes the sum of the squared *logarithmic* population deviations). That the effective rotational temperatures for all four combinations of surfaces and stretching modes lie well below the surface temperature of 473 K and implies a lack of equilibration between molecular rotation and the surface degrees of freedom, offering further evidence against a trapping-desorption scattering mechanism.

**TABLE 2:** Extracted mean rotational energies $\langle E_{rot.}^{\nu_{a}}\rangle$ and effective rotational temperatures $T_{rot.}^{\nu_{a}}$ from the rotational distributions presented in Figure 4. Rotational distributions were measured twice for Au(111), and we report the values of $\langle E_{rot.}^{\nu_{a}}\rangle$ and $T_{rot.}^{\nu_{a}}$ obtained from the run with the higher signal-to-noise ratio. The uncertainty of $\pm 5{cm}^{-1}$ reported for the Au(111) $\langle E_{rot.}^{\nu_{a}}\rangle$ values was obtained by taking the larger of the two deviations in $\langle E_{rot.}^{\nu_{a}}\rangle$ measured for the two runs. The reported uncertainties for $T_{rot.}^{\nu_{a}}$ are the standard errors reported by the least-squares fitting procedure implemented in the **optimize.curve_fit** function of the **SciPy** Python library (Virtanen et al., 2020).

Surface	$\langle E_{rot}^{\nu_{1}}\rangle$ [cm^-1^]	$\langle E_{rot}^{\nu_{3}}\rangle$ [cm^-1^]	$T_{rot.}^{\nu_{1}}$ [K]	$T_{rot.}^{\nu_{3}}$ [K]
Ni(111)	83.7	69.8	88±12	69±8
Au(111)	103±5	51±5	127±21	58±18

Perhaps the most interesting result emerging from this analysis is that the ratio of the degree of rotational excitation for molecules scattered into the $\nu_{1}$ and $\nu_{3}$ states differs dramatically for scattering from Au(111) versus Ni(111). The ratio of the mean rotational energies $\langle E_{rot}^{\nu_{1}}\rangle /\langle E_{rot}^{\nu_{3}}\rangle$ is 71% higher for Au(111) than for Ni(111). Similarly, the ratio $\langle T_{rot}^{\nu_{1}}\rangle /\langle T_{rot}^{\nu_{3}}\rangle$ is 72% higher for Au(111) than for Ni(111).

Why do the ratios $\langle E_{rot}^{\nu_{1}}\rangle /\langle E_{rot}^{\nu_{3}}\rangle$ and $\langle T_{rot}^{\nu_{1}}\rangle /\langle T_{rot}^{\nu_{3}}\rangle$ differ so dramatically for the two surfaces? We begin by pointing out that the $\nu_{3}$ fundamental lies $103\;{cm}^{-1}$ in energy above the $\nu_{1}$ fundamental (Boudon et al., 2006). A more general question thus poses itself: where does this extra vibrational energy go during an SIVR process? Since we cannot measure the speed of the scattered molecules or directly detect energy transfer to the surface, a complete experimental accounting of the energy balance is not possible. Nonetheless, our observations indicate that a greater portion of this excess energy is transferred to rotation in a collision with Au(111) $\left(\langle E_{rot}^{\nu_{1}}\rangle -\langle E_{rot}^{\nu_{3}}\rangle =52\;{cm}^{-1}\right.$ ) than is transferred in a collision with Ni(111) ( $\langle E_{rot}^{\nu_{1}}\rangle -\langle E_{rot}^{\nu_{3}}\rangle =14\;{cm}^{-1}$ ).

In the reduced-dimensional CH_4_/Ni(111) SIVR calculations presented in *Werdecker et al.*, the surface was held frozen and the molecule was forced to assume at all times the minimum energy orientation so that energy released in the $\nu_{3}\to \nu_{1}$ conversion was constrained to be transferred completely into translational kinetic energy of the scattering molecule. In light of our results, it would be of keen interest to learn how this energy is partitioned in a dynamical simulation that includes both molecular rotation and surface vibrations as degrees of freedom. It would be equally interesting to learn to what extent the catalytic C-H bond elongation discussed in Introduction is also operative on Au(111). Perhaps the enhanced $\nu_{1}$ rotational excitation on Au(111) reflects a mechanism for SIVR on Au(111) that is fundamentally different from the catalytic effect thought to be operating on Ni(111). SIVR on Au(111) might, for example, be a purely “mechanical” effect arising from the violent recoil of the molecule experiences upon rebounding from the much more massive gold surface atoms. An influence of the surface atom mass on CH_4_/metal scattering inelasticity has been observed in recent dynamical calculations (Gerrits et al., 2019; Jackson, 2022) which finds Baule-like molecule–surface energy transfer in CH_4_/metal surface collisions. A measurement of CH_4_/Cu (111) SIVR efficiency might shed light on the role of a mechanical mechanism. The catalytic activity of Cu(111) with a calculated barrier height of 1.32 eV (Bhati et al., 2022) falls in between that of the Ni(111) and the Au(111) surface. In light of both this and the ability of the lighter copper surface atoms to better absorb the shock of impact, a Cu(111) branching ratio weaker than that of Au(111) would stand as strong evidence for a mechanical SIVR mechanism operating on Au(111).

The last observation we wish to make about the rotational distributions concerns the $\nu_{3}$ levels of equal J , a subject we began discussing at the end of the previous section (Section 3.2) in the context of angular distributions. Figures 4, 5 clearly show that scattering from the Au(111) produces a highly imbalanced distribution of populations among $\nu_{3}$ sublevels of equal J . These imbalances imply a scattered molecular flux with a high degree of both internal alignment of the total angular momentum (indicated by the imbalance of the $A_{1}$ and $A_{2}$ sublevel populations within the $\left(\nu_{3},J=5,F^{\left(+\right)}\right)$ Coriolis stack) and relative alignment of the total and vibrational angular momentum (indicated by imbalances among the Coriolis stacks of equal J for J=3,4,5 ). From the restricted subset of levels tagged in this experiment, there appears to be a strong tendency for preservation in the CH_4_/Au(111) scattering event of the parallel (i.e., $F^{\left(-\right)}$ ) alignment of total and vibrational angular momentum prepared by the pump laser. Although the $\nu_{3}$ population imbalances within a given J are not as pronounced in the CH_4_/Ni(111) scattering data, it is nonetheless interesting to note that the order of the imbalances for the two surfaces are identical for the levels probed.

At the present time, the authors have no explanation for the form and extent of this internal alignment of molecular rotation observed in the scattering. We plan to present more extensive results on these interesting alignment phenomena in CH_4_ surface scattering in a future publication.

## 4 Summary and future directions

A number of promising avenues exist for further testing the connection between SIVR and catalytic activity. One extension of the work presented here would be a study of SIVR efficiency for a range of different incident kinetic energies $E_{i}$ . In one sense, one expects SIVR to correlate positively with $E_{i}$ since, with the increasing incident energy, the molecule can get closer to the surface and gain access to the catalytic forces that distort the molecule’s equilibrium geometry and mediate vibrational mode coupling. Indeed, a positive $E_{i}$ -SIVR correlation is observed in the CH_4_/Ni (111) dynamical calculations of *Werdecker et al.* discussed in Introduction. On the other hand, depending on the form of the repulsive molecule–surface interaction, a high-incident velocity may result in the brief molecule–surface interaction time, which might tend to weaken the SIVR produced on scattering.

Another direction, one which would offer a more direct validation of the theoretical results reported in *Werdecker et al.*, would be to measure the variation in SIVR across the different microscopic impact sites of the same macroscopic surface. Although it is of course not feasible to experimentally control the impact site of a molecule, one can vary the relative concentration of different sites. In the calculations of *Werdecker et al.*, the authors find an increase in SIVR at sites where a surface Ni atom is displaced from equilibrium. Such displacements are also expected to increase a site’s catalytic activity (Nave et al., 2010). By raising/lowering the surface temperature, one can increase/decrease the density of thermally generated lattice distortions and test for the expected increase/decrease in SIVR.

Another more controlled means of varying the density of different surface sites is to vary the exposed crystal plane, characterized by Miller indices (*ijk*) (Kolasinski, 2012). For *fcc* metals (e.g., Ni, Au, and Pt), the atoms of the (111) (or “close-packed”) surface are highly coordinated and therefore present sites of relatively low catalytic activity. The stepped *fcc*(211) surface, however, exposes under-coordinated atoms at step sites, offering lower barrier sites for methane dissociative chemisorption, as observed in experiments and calculations for CH_4_ sticking experiments on platinum (Chadwick et al., 2018). A measurement of the branching ratios for different surface planes of the same crystal could thus serve as a compelling experimental verification of the theoretically observed connection between a site’s SIVR efficiency and its catalytic activity.

We conclude with a brief summary. For the Ni(111) and Au(111) surfaces, we measured rovibrational state-resolved angular distributions for 10 (4) rotational levels of the $\nu_{3}$ ( $\nu_{1}$ ) stretch fundamentals produced by scattering of incident CH_4_( $\nu_{3},J=1$ ) molecules. From these distributions, we extracted for each surface a branching ratio $br_{\nu_{1}/\nu_{3}}$ characterizing the efficiency of collision-induced $\nu_{3}\to \nu_{1}$ conversion. The branching ratio measured for the Au(111) surface is found to be more than five times smaller than that measured for the Ni(111) surface but significantly larger than that measured by *Werdecker et al.* at a fixed scattering angle for the Gr/Ni(111) surface. The ordering of the branching ratios for the three surfaces is found to match the ordering of their catalytic activities, which is in accordance with the hypothesis advanced in *Werdecker et al*.

Analysis of the shape of angular distributions reveals for both surfaces a broadening of the scattering distributions with increasing total angular momentum quantum number J , with the broadening stronger for scattering from the Au(111) surface. In addition, we observe a tendency for super-specular scattering, and this tendency increases with J and is more pronounced for the Au(111) surface. For the Ni(111) surface, the elastic channel is strong and anomalously narrow. The rovibrational levels of equal J in the $\nu_{3}$ stretch show measurable differences in width and peak angle in scattering from the Ni(111) surface.

From the distribution of scattering angle-integrated state fluxes, the Ni(111) surface produces more highly rotationally excited molecules in the $\nu_{3}$ stretch than the Au(111) surface. Molecules in the $\nu_{1}$ stretch are, however, found to be dramatically more rotationally excited in scattering from Au(111) than from Ni(111), prompting a questioning into the nature of the flow of vibrational energy for the two surfaces. The rovibrational levels of equal J in the $\nu_{3}$ stretch have strongly imbalanced populations upon scattering from Au(111), suggesting strong alignment of the internal angular momentum in the scattered flux and a preservation of the parallel Coriolis coupling of the total and vibrational angular momentum prepared in the incident molecules.

## Data Availability

The original contributions presented in the study are included in the article/Supplementary Material. Further inquiries can be directed to the corresponding author.

## Footnotes

1 Jackson, B. (2023) Private communication.
