# Supplementary material for: Surface-induced vibrational energy redistribution in methane/surface scattering depends on catalytic activity
Source: Front Chem. 2023 Jul 25;11:1238711. doi: 10.3389/fchem.2023.1238711 (PMC10426747; doi:10.3389/fchem.2023.1238711)
Supplement: Supplementary file 1 [file DataSheet1.pdf]

Supplementary Materials

Patrick Floß[1,2], Christopher S. Reilly[1], Daniel J. Auerbach[1,3] and Rainer D. Beck[1, *]

[1] Institute of Chemical Sciences and Engineering (ISIC), Group for Gas-Surface Dynamics, École Polytechnique Fédérale de Lausanne (EPFL), CH-1015 Lausanne, Switzerland

[2] Max Planck-EPFL Center for Molecular Nanoscience and Technology, Göttingen, Germany and Lausanne, Switzerland

[3] Max Planck Institute for Multidisciplinary Sciences, D- 37077 Göttingen, Germany

Correspondence:
Corresponding Author
rainer.beck@epfl.ch

S1. State-resolved in-plane angular distributions

Figures S1-S2 show the 28 state-resolved angular distributions obtained for scattering of CH_4 ($\nu_3, J = 1, F^{(-)}, A_2, \alpha = 3$) with E_i = 100 meV, θ_i = 35° and T_S = 473K from Ni(111) (Figs. S1a and S1b) and Au(111) (Figs. S2a and S2b). Experimental scattering intensity is shown as points and the best fit of equation 1 (in main article) to the data as dashed lines (see graphs for best-fit parameters). For each surface we probed 14 rovibrational states. The tagged state is given above each state-resolved angular distribution. For the identification of the probed level, we use the same nomenclature used in the main article.

The probed levels for one surface are organized as follows in the supplementary figures: States resulting from vibrationally inelastic scattering are always shown in the right column, vibrationally elastic scattering is shown in both columns. Additionally, the rotational quantum number increases from the top of each panel to the bottom.

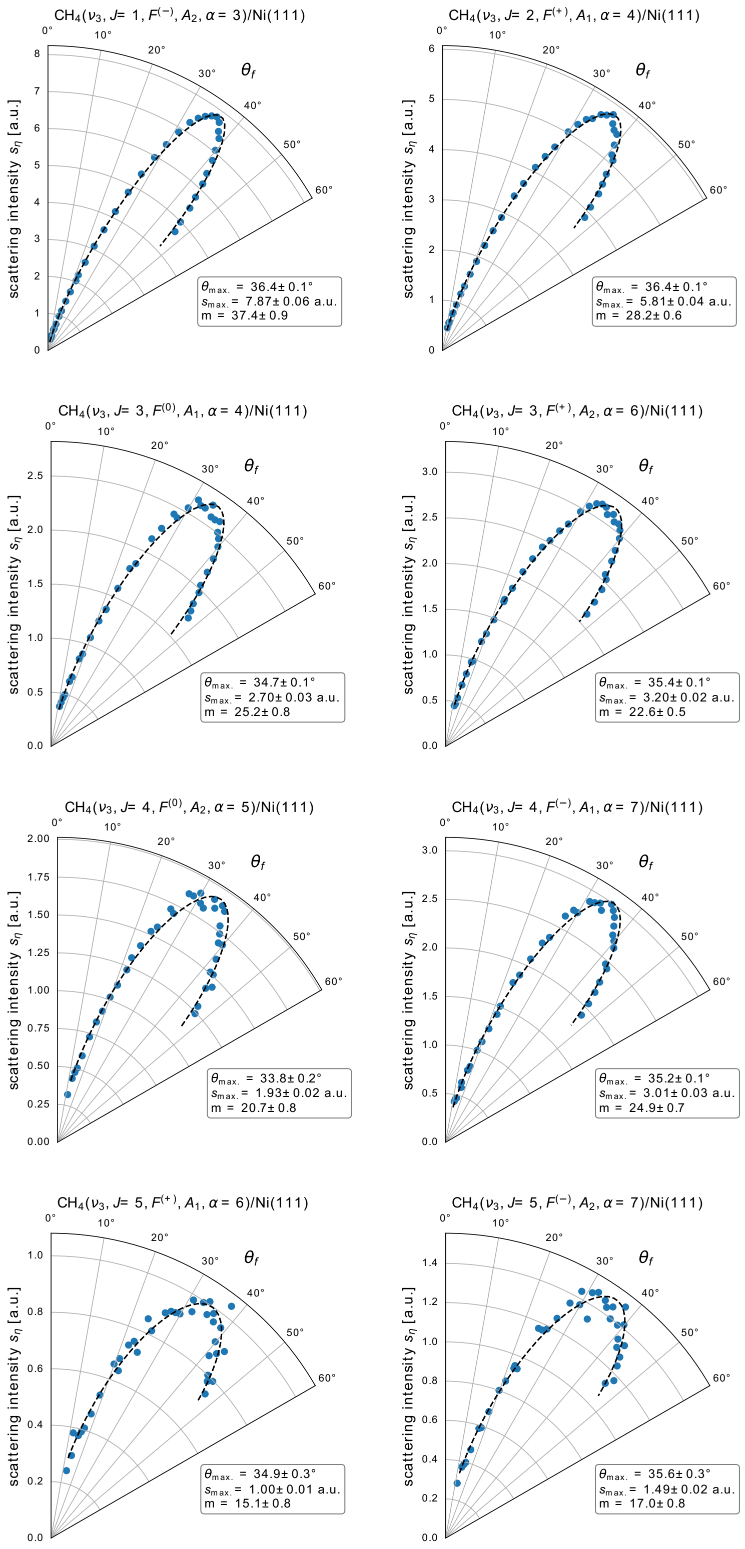

Figure S1a: State-resolved in-plane angular distributions $s_\eta(\theta_f)$ for $CH_4(\nu_3, J = 1, F^{(-)}, A_2, \alpha = 3)$ scattering from Ni(111). The incoming molecules have a mean kinetic energy $E_i = 100 \pm 13$meV and strike the surface ($T_s = 473$K) at an incident angle $\theta_i = 35°$.

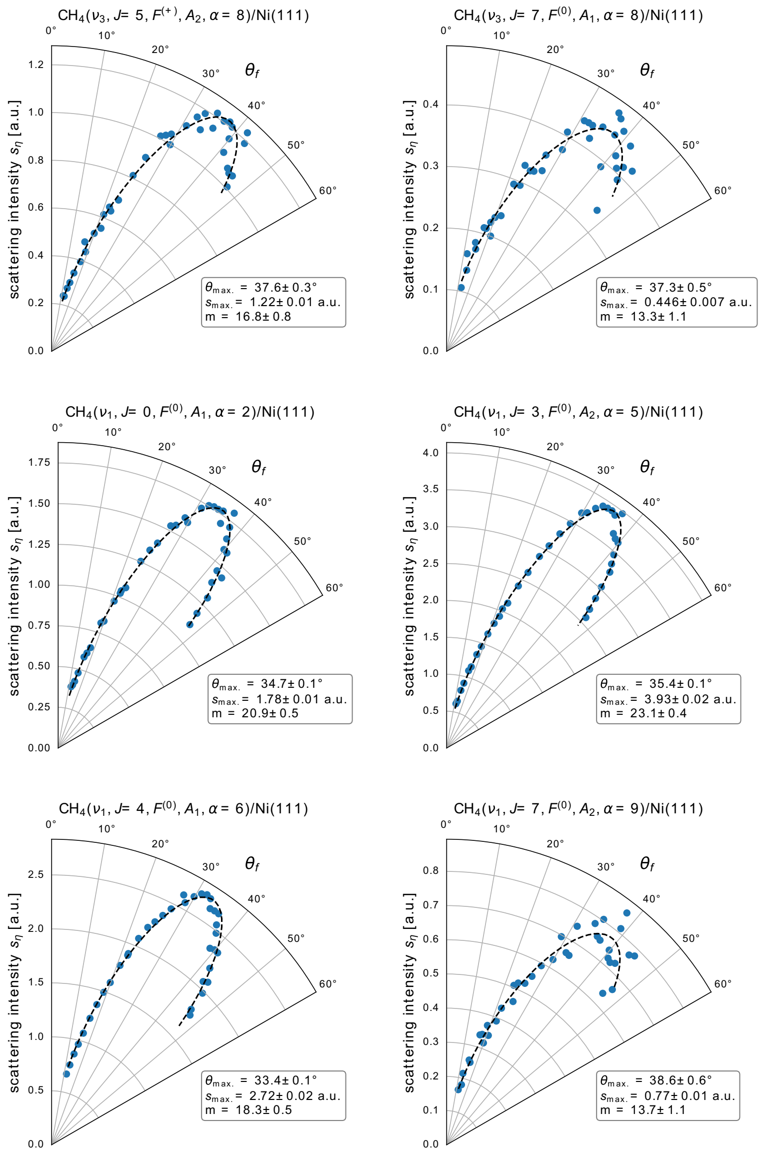

Figure S1b: State-resolved in-plane angular distributions $\boldsymbol{s_\eta(\theta_f)}$ for $CH_4(\boldsymbol{\nu_3, J=1, F^{(-)}, A_2, \alpha=3})$ scattering from Ni(111). The incoming molecules have a mean kinetic energy $\boldsymbol{E_i = 100 \pm 13}$meV and strike the surface ($\boldsymbol{T_s = 473}$K) at an incident angle $\boldsymbol{\theta_i = 35}$°.

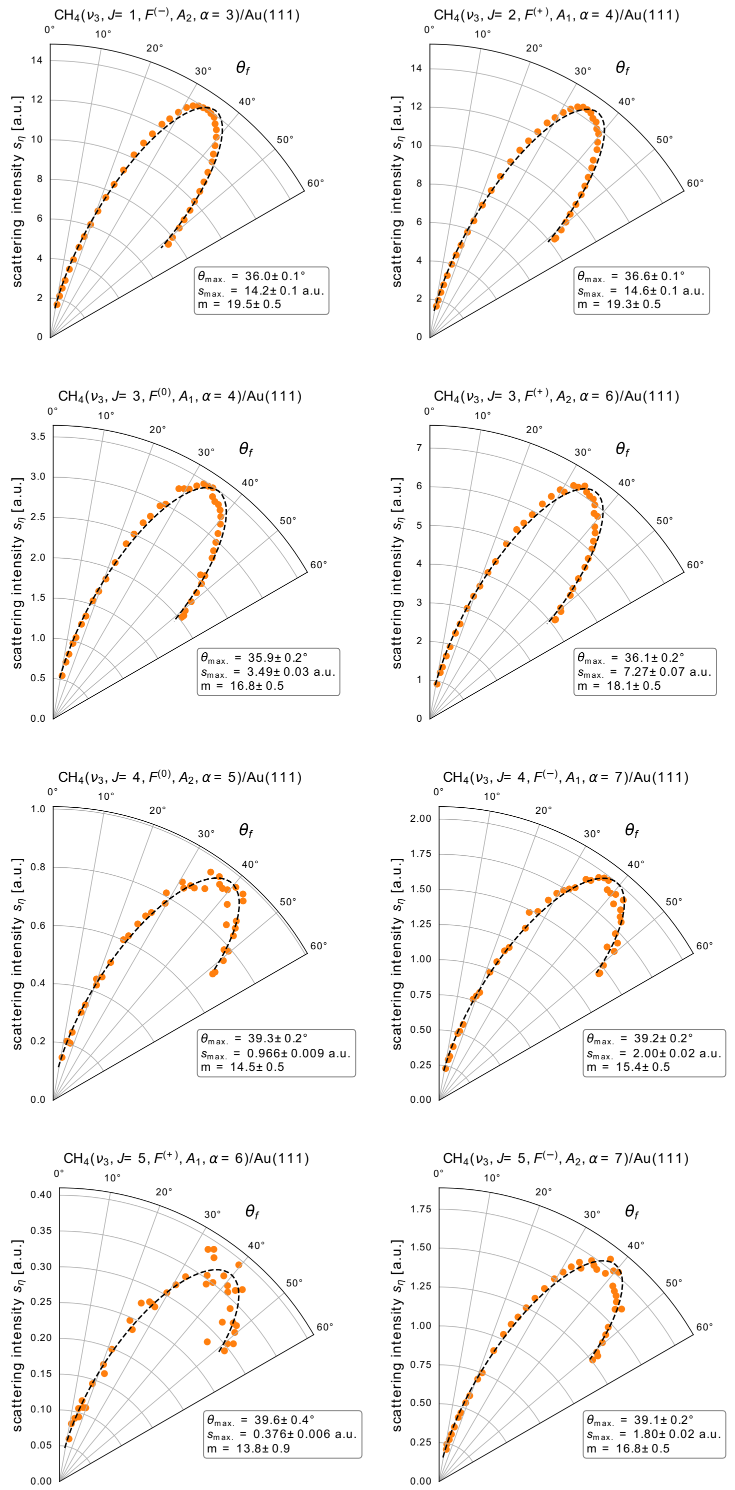

Figure S2a: State-resolved in-plane angular distributions $s_\eta(\theta_f)$ for CH4($\nu_3, J = 1, F^{(-)}, A_2, \alpha = 3$) scattering from Au(111). The incoming molecules have a mean kinetic energy $E_i = 100 \pm 13$meV and strike the surface ($T_s = 473$K) at an incident angle $\theta_i = 35°$.

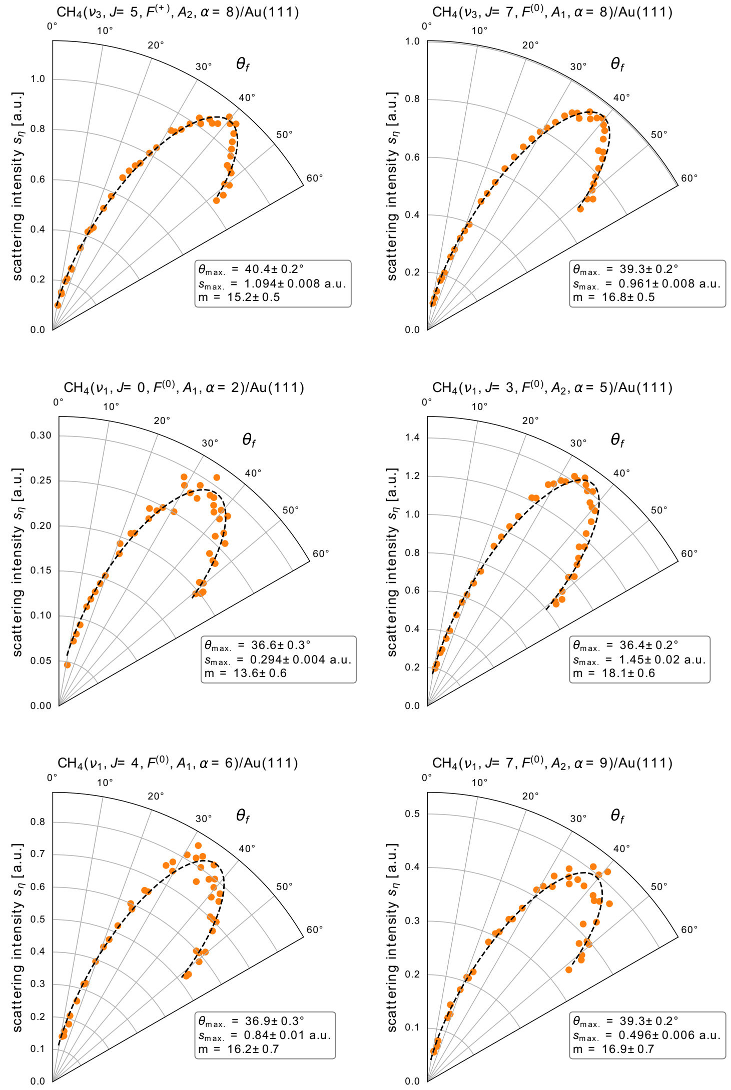

Figure S2b: State-resolved in-plane angular distributions $\boldsymbol{s_\eta(\theta_f)}$ for $CH_4(\boldsymbol{\nu_3, J=1, F^{(-)}, A_2, \alpha=3})$ scattering from Au(111). The incoming molecules have a mean kinetic energy $\boldsymbol{E_i = 100 \pm 13}$meV and strike the surface ($\boldsymbol{T_s = 473}$K) at an incident angle $\boldsymbol{\theta_i = 35}$°.

S2. Scattering angle resolved branching ratio and scattering intensity

We have already shown in the main article (see Fig. 2 & 3) and the figures S1-S2 that the angular distributions differ significantly for the different probed levels. We thus argued that an integration over the widest angular range in plane (and ideally also out-off plane) is important to obtain an accurate measurement of the branching ratio. To illustrate the importance of this effort, we calculate the branching ratio for each individual scattering angle, as if we only had an apparatus with a fixed geometry. For each vibrational mode by we first calculate the total scattering intensity at a scattering angle by: $s_{\nu_a}(\theta_f) = \sum_{\eta\in\nu_a} s_\eta(\theta_f)$. The branching ratio at this fixed scattering angle is thus calculated as $\mathrm{b}r_{\nu_1/\nu_3}(\theta_f) = s_{\nu_1}(\theta_f)/s_{\nu_3}(\theta_f)$ and shown as points in Fig. S3, "data". Alongside with the points we also show a dashed line marked as "fit" in the legend. For this "fit" we used the best-fit parameters describing each angular distribution such that we calculate the scattering intensity at a fixed scattering angle as:

$$s_\eta(\theta_f) = s_\eta^{fit}(\theta_f) = s_{\mathrm{max.}(\eta)} \cdot cos^{m(\eta)}(\theta_{\mathrm{max.}(\eta)} - \theta_f).$$

Additionally, we compute the scattering intensity $\Omega(\theta_f)$ at each scattering angle in % per degree to illustrate the weight of the branching ratio at a fixed scattering angle towards the total branching ratio as:

$$\Omega(\theta_f) = \frac{s_{\nu_1}(\theta_f) + s_{\nu_3}(\theta_f)}{\bar{S}_{\nu_1} + \bar{S}_{\nu_3}}$$

We visualize the above calculated scattering intensity with a colormap in the Fig. S3. In the main article we already pointed out the narrowness of the elastic scattering from Ni(111). We assumed this feature originates from a scattering trajectory with a turning point farther away from the surface and hence a minor probability to undergo rotational and/or vibrational inelastic scattering.

Sticking with this idea it would thus be logical to also compute a branching ratio for those molecules scattering only inelastically from the surface, sharing a scattering trajectory turnaround closer to the surface. For Ni(111) this inelastic branching ratio is 46.3% and for Au(111) it is 9.6%, compared to the 35.1% and 6.6% when including the elastic scattering channel. In Fig. S3 we also show the branching ratio at fixed scattering angles based on the inelastic scattering channels by squares ("inelastic").

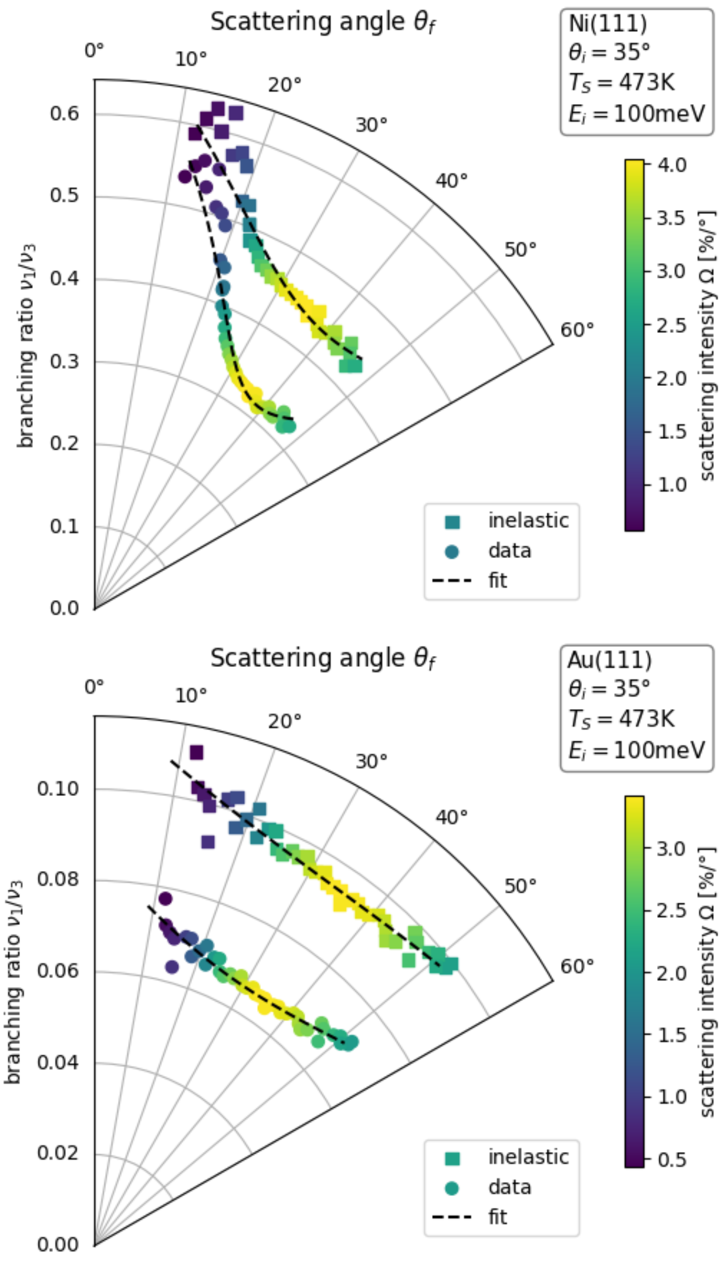

Figure S3: Branching ratio $br_{\nu_1/\nu_3}(\theta_f)$ and scattering intensity $\Omega(\theta_f)$ shown as a function of scattering angle (θ_f), deduced from state-resolved in-plane angular distribution measurements of $CH_4(\nu_3, J = 1, F^{(-)}, A_2, \alpha = 3)$ scattering from Ni(111) (top panel) and Au(111) (bottom panel). The incoming molecules have a mean kinetic energy $E_i = 100 \pm 13$meV and strike the surface ($T_s = 473$K) at an incident angle $\theta_i = 35°$.

From Figure S3, we can clearly see how the branching ratio varies for different scattering angles, highlighting the importance to measure the angular distributions. This variation is particularly important for scattering from Ni(111). For both surfaces neglecting the elastic channel changes the magnitude of the branching ratio, but only for scattering from Ni(111) does the elastic channel have a pronounced influence on the shape of the angular distribution.
